# Luminescence-Based Optical Sensors Fabricated by Means of the Layer-by-Layer Nano-Assembly Technique

**DOI:** 10.3390/s17122826

**Published:** 2017-12-06

**Authors:** Nerea De Acha, Cesar Elosua, Ignacio Matias, Francisco Javier Arregui

**Affiliations:** 1 Department of Electric and Electronic Engineering, Public University of Navarra, E-31006 Pamplona, Spain; cesar.elosua@unavarra.es (C.E.); natxo@unavarra.es (I.R.M.); parregui@unavarra.es (F.J.A.); 2 Institute of Smart Cities (ISC), Public University of Navarra, E-31006 Pamplona, Spain

**Keywords:** photoluminescence, layer-by-layer nano-assembly technique, nanostructured materials, chemical sensing

## Abstract

Luminescence-based sensing applications range from agriculture to biology, including medicine and environmental care, which indicates the importance of this technique as a detection tool. Luminescent optical sensors are required to be highly stable, sensitive, and selective, three crucial features that can be achieved by fabricating them by means of the layer-by-layer nano-assembly technique. This method permits us to tailor the sensors′ properties at the nanometer scale, avoiding luminophore aggregation and, hence, self-quenching, promoting the diffusion of the target analytes, and building a barrier against the undesired molecules. These characteristics give rise to the fabrication of custom-made sensors for each particular application.

## 1. Introduction

Optical sensing techniques allow for the possibility of making remote [1] and non-invasive measurements [2], as well as working in hazardous environments [3]. These, and many other significant advantages over other detection technologies, have attracted the attention of scientists over the last decades [4,5], and hence this technology has experienced a high development.

Optical sensors can be based on different transduction mechanisms, such as absorption [6,7], resonance [8,9,10], or photoluminescence [11,12,13]. The latter consists of emission of light by a material as a consequence of its previous absorption at lower wavelengths (excitation). Depending on the lifetime of this emission (i.e., the average time it takes the intensity to drop by 1/*e*), luminescence can be classified as fluorescence (lifetime in the range of ps and ns) or phosphorescence (lifetime greater than ms). The intensity of this emission, as well as its lifetime, can be quenched or enhanced by the variation of different external parameters: pH [14], temperature [15], biomolecules [16], oxygen [17], or metal ion concentration [18]. This modulation of the intensity (and lifetime) by external parameters has been widely employed for the development of luminescence-based sensors, either in solution [19,20], or onto different substrates [21,22,23].

Among the existing luminescent materials (also known as luminophores), quantum dots (QDs) [24,25], nanoparticles (NPs) [26,27], fluoropolymers [28], dyes [29,30], and porphyrins [31,32,33] have been the most utilized. For the fabrication of sensors, these luminescent materials are usually entrapped or encapsulated in different matrices [34,35] or shells [36,37], which must be designed to facilitate the interaction between the analyte and the sensing material [38].

There are three key requirements that luminescent sensors must meet: good photostability, and high selectivity and sensitivity [39]. To achieve these characteristics and allow the rapid adsorption/desorption of the target analytes to the sensing films, highly permeable coatings are usually fabricated by means of dip-coating [40], spin-coating [41], sol-gel [42], or xero-gel [43] techniques. However, when utilizing these methods the distribution of the luminophore inside the films cannot be controlled, which gives rise to their aggregation and causes self-quenching, hence significantly reducing the sensors′ sensitivity [44]. This can be overcome by fabricating the sensing coatings by means of the layer-by-layer nano-assembly (LbL) technique, which consists of the deposition of oppositely charged materials (typically polyelectrolytes) by electrostatic forces or other attractive forces acting cooperatively, including interactions such as hydrophobic attraction [45]. LbL has been experimentally demonstrated to be a powerful method for the fabrication of luminescence-based sensors, since it is a reproducible technique that allows the utilization of a wide variety of indicators. Furthermore, an accurate selection of materials and assembly parameters not only permits us to modify the permeability of the nanostructure [46], hence promoting the diffusion of target species or forming a barrier against undesirable ones [47], but also allows us to control the layer thickness at the nanometer scale and tailor the space distance between luminescent layers [48]. Taking advantage of this fact, it is possible to tailor the homogeneity of the distribution of the luminophore into the matrix [49] in order to attenuate self-quenching [48].

In recent years, luminescence has become a powerful detection mechanism in a broad range of areas, being the most important sensing tool in different biological applications [50]. This fact, together with the versatility that the LbL technique offers for the fabrication of custom-made sensors [51], has led to the development of a wide variety of luminescent probes built by this technique. Thus, a review in which the principal luminescence-based sensors fabricated by the LbL technique are analyzed is of great interest. This review compiles solution probes as well as multilayer sensing films for different purposes: metal ions detection, dissolved and gaseous oxygen monitoring, and biosensing applications.

## 2. Luminescent Sensors Based on Encapsulated Indicators

The progress of in vivo measurement systems in recent years has yielded the development of biocompatible optical sensors [52]. A widespread method for the fabrication of this kind of sensor consists of the encapsulation of the sensitive material in multilayered nanostructures (also called shells or capsules). These walls have to meet a key requirement: protecting sensitive molecules from the external environment while allowing fast diffusion of the target analyte [53]. LbL encapsulation of sensors was first described in the early 2000s [54,55] and, since then, many different applications have been reported [56,57,58,59].

**Figure 1:**
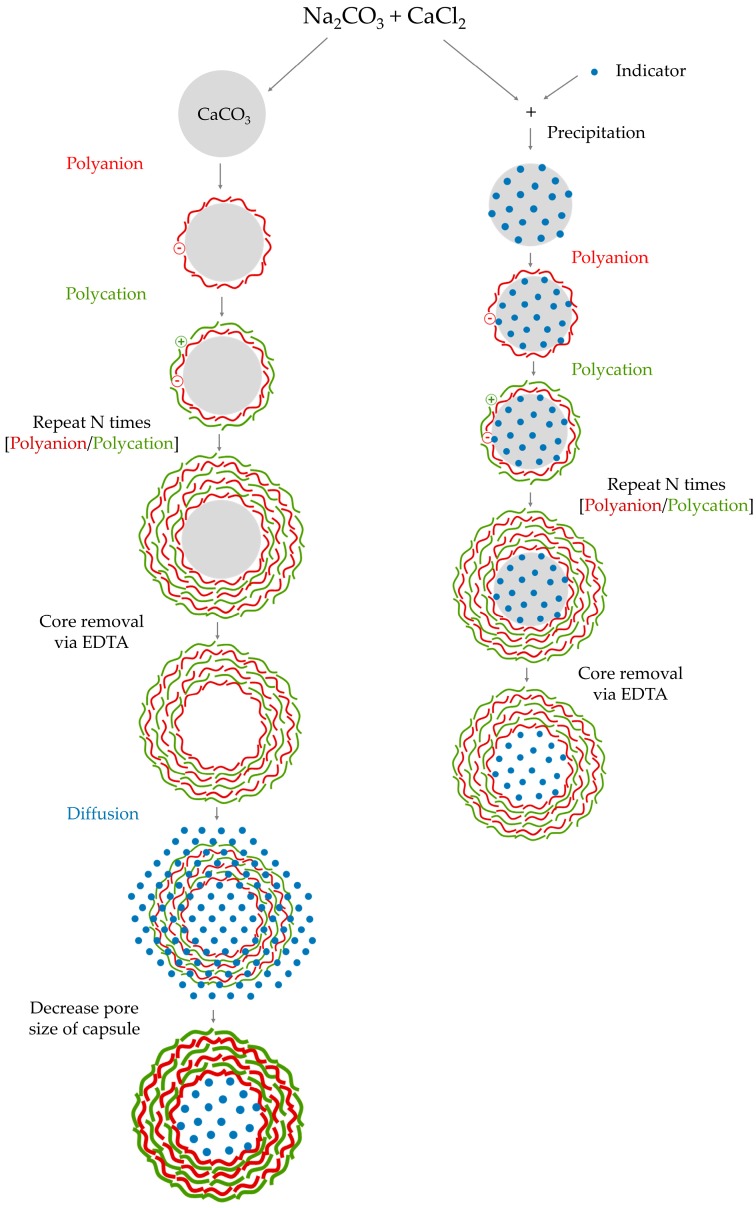
Schematic of the encapsulation techniques.

Particle encapsulation requires a template that is coated with a multilayered nanostructure and then dissolved. The main methods for immobilizing the luminophores inside the shells are diffusion and precipitation [60], which are illustrated in Figure 1.

In the case of encapsulating the sensing molecules by diffusion, the multilayered coatings are adsorbed onto sacrificial templates, usually dissolvable or degradable inorganic polymeric microspheres. Once the capsules are fabricated, the templates are dissolved in order to leave hollow microspheres suspended in solutions of the sensing molecules, which are loaded inside them just by diffusion [61]. Despite this technique offering the possibility of employing almost any dissolvable template, it also exhibits the lowest loading efficiency.

When the luminophores are encapsulated by precipitation, they co-precipitate with the sacrificial templates before the shell is built and, subsequently, templates are diluted [58]. By employing this technique, the highest loading efficiency rates can be obtained.

For an adequate selection of templates, it is important to take into account two factors: firstly, they must be able to keep their structural properties during the coating process and, secondly, they have to be easily dissolved once the encapsulating nanostructure has been attached to their surface [58]. Typical materials used as templates are calcium carbonate (CaCO_3_) [62], silica [63], latex [64] polystyrene [65], or gold [66] nanoparticles and melamine formaldehyde [67] microspheres. The dimensions of the shell depend on their shape and size, which can range from nanometers to microns [68].

Owing to the versatility of the LbL method, it has been extensively used for micro- and nanoparticle encapsulation [69,70], usually employing polyelectrolytes. That is because, by varying some properties of polyelectrolyte solutions and controlling the deposition conditions, it is possible to tailor the properties of the capsule walls [71]. For instance, the pH of the solutions not only influences the shell thickness [72], but also its permeability [73] and, hence, the diffusion rate of molecules inward.

As mentioned below, capsules are usually made of different polyelectrolyte combinations. The most used pairs are poly(allylamine hydrochloride) (PAH)/poly(styrene sulfonate) (PSS) [74] and poly(diallyldimethylammonium chloride) (PDDA)/poly(styrene sulfonate) (PSS) [75]. Apart from them, other combinations of materials can be employed, for instance, poly-l-lysine/poly(l-glutamatic acid) [76], chitosan/dextran [63], or poly-l-lysine/heparin [77]. However, their use is not as common as the first mentioned polyelectrolyte pairs.

One of the advantages of this technique is the possibility of developing self-referenced sensors by using a sensitive luminophore and a non-sensitive luminophore that acts as an optical reference [63]. Ideally, both have the same absorption spectrum and a complementary emission spectrum [52], which allows the use of a single excitation light source and the simultaneous monitoring of the emission peaks: one of them will change in the presence of the target analyte, while the other will remain constant. Apart from encapsulating both luminophores [58], it is also common that one of them is assembled as part of the multilayered coating [78].

The main applications of these sensors (metal ion detection, dissolved oxygen sensing, and glucose and lactate monitoring) are analyzed in the following subsections.

### 2.1. Encapsulated Sensors for Ion Detection

Metal ions are known to be highly toxic materials [79], so considerable efforts have been made towards their detection in aqueous media. Duchesne and co-workers [80] fabricated a potassium sensor by encapsulating the fluorescent indicators by the diffusion technique: using positively charged melamine formaldehyde (MF) particles as templates, multilayer capsules of PSS and PDDA were fabricated. The MF cores were diluted afterwards in HCl, and then the capsules were immersed in solutions of potassium-binding benzofuran isophthalate tetraammonium salt (PBFI), which diffused through the walls to the hollow cores. The PBFI-loaded sensors exhibited a luminescent peak at 500 nm, which moved towards lower wavelengths and increased in intensity upon the addition of K^+^ ions in the 0–45 mM range. 

**Figure 2:**
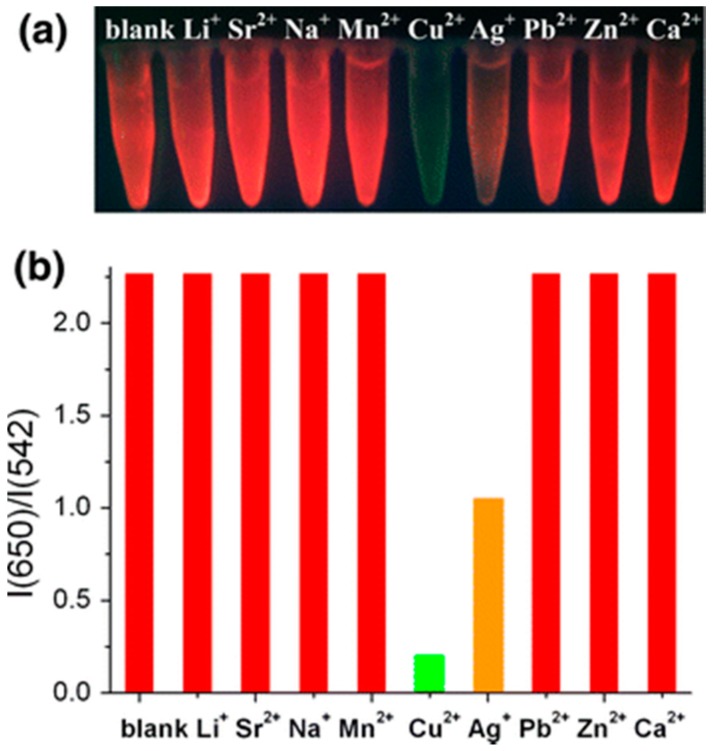
Effect of the different metal ions on the RE–QD composites: (**a**) Cu^2+^ and Ag^+^ ions change the color of the RE-QD composites into green and orange, respectively; (**b**) The ratio I(650)/I(542) of the RE-QD composites only decreases in presence of Cu^2+^ and Ag^+^. Reprinted from [83] with permission from Springer.

Rare-earth (RE) nanocrystals are of great interest for sensing applications because of their optical and chemical properties, such as long lifetime (in the range of μs or ms) [81], high quantum yield, or resistance to photobleaching [82]. A fluorescent sensing system based on RE nanocrystals and CdSe/ZnS quantum dots was fabricated by means of the LbL technique in [83]: PEI-coated NaYF_4_:Ce,Tb nanorods were separated from the CdSe/ZnS QDs by a PSS/PAH bilayer. Under UV illumination (255 nm), a dual emission was observed, with the luminescent peaks centered at 542 nm (RE nanocrystals) and 650 nm (QDs), with the latter being dominant due to the fluorescence resonance energy transfer between the RE nanorods and the QDs. Under exposure to different concentrations of Cu^2+^ or Ag^+^ metal ions in the μM range, the luminescent intensity at 650 nm decreased, while that corresponding to the peak centered at 542 nm remained constant. This can be observed in Figure 2, in which the system was exposed to different metal ions (30 μM): the red color (650 nm) was quenched by Ag^+^ ions, while it completely disappeared in the presence of Cu^2+^ ions, demonstrating high selectivity.

Other encapsulated sensors for the detection of metal ions are summarized in Table 1. Most of them use salts as sensitive indicators (e.g., benzofuran isophthalate tetraammonium salt (PBFI) or sodium-binding benzofuran isophthalate tetraammonium salt (SBFI)), and FluoSpheres as references.

### 2.2. Encapsulated Sensors for Dissolved Oxygen Detection

The most important application of this kind of sensors is the detection of low concentrations of dissolved oxygen for biomedical applications, typically utilizing ruthenium porphyrins as indicators. McShane et al. [52] fabricated a self-referred oxygen sensor by employing tris(2,2′-bipyridyl) dichlororuthenium(II) hexahydrate (Ru(bpy)) as the sensitive material and fluorescein isothiocyanate (FITC) as the reference one, so the use of a single light source at 460 nm was possible, and the simultaneous monitoring of the luminescent peaks at 525 nm (FITC) and 620 nm (Ru(bpy)), allowing ratiometric measurement of the fluorescence (I_620_/I_525_). For the capsule fabrication, 2.6 µm melamine formaldehyde microtemplates were coated with {PSS/PAH-FITC}_n_ or {PSS/PAH-FITC}_n_PSS shells. After the cores’ dilution, the capsules were suspended in solutions of the oxygen-sensitive indicator at different pH values, and Ru(bpy) molecules were loaded by diffusion. The highest loading efficiency was achieved in the case of the {PSS/PAH-FITC}_5_PSS shells, when the pH value of the Ru(bpy) solution was 10.4. Suspensions of these sensors were bubbled with O_2_ and N_2_, and it was observed that in the presence of O_2_, the intensity at 620 nm (Ru(bpy)) decreased while the intensity at 525 nm (FITC) remained constant. A total decrease of 15% of the I_620_/I_525_ ratio was measured when only O_2_ was bubbled. Moreover, the sensor response was consistent with the dynamic changes of the gas levels over time.

**Figure 3:**
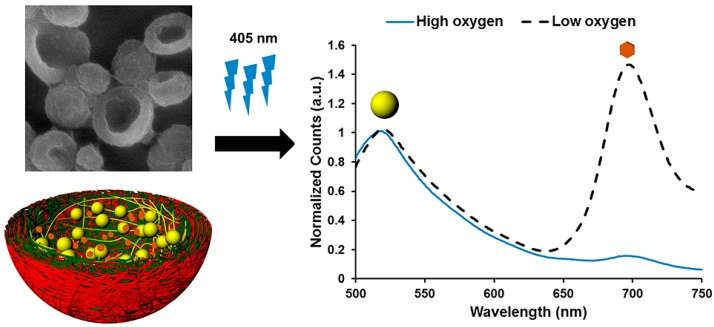
SEM image of the nano-capsules after the dilution of the core (**upper left**), schematic representation of the encapsulated sensors (**lower left**) and luminescence spectra of the sensors when exposed to high and low dissolved oxygen concentrations (**right**). Reprinted with permission from [58]. Copyright: 2014, American Chemical Society.

Palladium porphyrin was encapsulated in [58] by the co-precipitation technique: the carboxylate modifies FluoSpheres (FS), used as the reference, and the Pd-meso-tetra(4-carboxyphenyl) porphine (PdTCPP) co-precipitated with the calcium carbonate (CaCO_3_) nanoparticles. They were first stabilized in poly(vinylsulfonic acid) (PVSA) and then coated with the multilayer (PDDA/PSS)_10_ structure, and the CaCO_3_ cores were diluted. When the capsules were excited at 405 nm, they emitted luminescence at 515 nm (FS) and 700 nm (PdTCPP). Under exposure to different dissolved oxygen concentrations, the intensity of the emission peak centered at 515 nm remained constant, while that of the peak at 700 nm decreased proportionally to the oxygen concentration. Thus, the ratio I_700_/I_515_ was used to characterize the sensor, which had a detection limit of 7.62 μM. A schematic illustration of this sensor and its response to oxygen is given in Figure 3.

Other dissolved oxygen probes were developed with different ruthenium porphyrins, such as tris(4,7-diphenyl-1,10-phenanthroline)ruthenium(II) dichloride (Ru(dpp)) or [Ru(Ph_2_phen)_3_]^2+^. They are summarized in Table 2.

### 2.3. Encapsulated Sensors for Glucose and Lactate Monitoring

**Figure 4:**
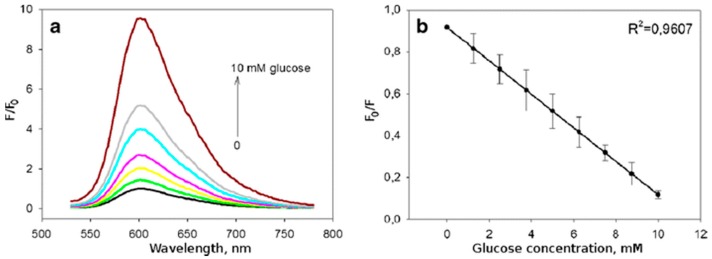
(**a**) Relative fluorescence intensity upon the addition of different glucose concentrations. F and F_0_ represent the fluorescence intensities in the presence (F) and absence (F_0_) of glucose; (**b**) calibration curve of the sensor. Reprinted from [87] with permission from Springer.

A particular application of dissolved oxygen sensors is glucose monitoring [89]. To this end, glucose binding proteins, usually glucose-oxidase [87] or apo-glucose-oxidase [90], are loaded inside the multilayered shells, as well as the sensitive molecules. For instance, Kazakova et al. [87] fabricated 5 μm-microcapsules made of PAH and PSS loaded with oxygen-sensitive dye (Ru(dpp)) that entrapped glucose-oxidase. This indicator emitted fluorescence between 560 and 700 nm, and its intensity was inversely proportional to the oxygen concentration. Furthermore, glucose concentration was correlated with oxygen reduction during enzymatic degradation by glucose oxidase. An increase in the fluorescent intensity and the calibration curve of this sensor upon the addition of different glucose concentrations is observed in Figure 4.

**Figure 5:**
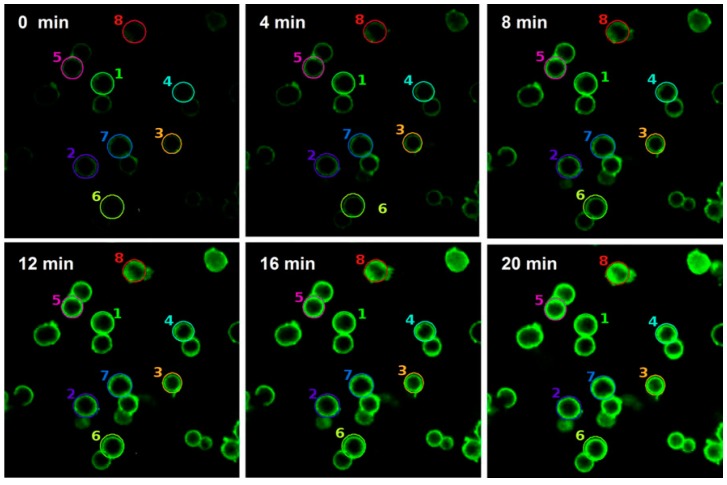
Сonfocal microscopy images of DHR123-labeled capsules containing lactate oxidase in the presence of 0.23 nM peroxidase and 4 mM lactate. Reprinted from [87] with permission from Springer.

Kazakova et al. also coated lactate oxidase and peroxide with capsules of PAH and PSS loaded with dihydrorhodamine 123 (DHR123), which was sensitive to hydrogen peroxide. The addition of lactate in the presence of lactate oxidase produced hydrogen peroxide, which oxidized DHR123 in the presence of peroxide, yielding rhodamine123, a molecule that emitted green fluorescence (510–560 nm), as can be observed in Figure 5.

## 3. Luminophores Immobilized in Multilayer Films for Sensing Applications

Luminescence-based sensors are also fabricated onto different substrates by coating them with films in which the sensitive luminophores are embedded. These luminescent coatings exhibit many different advantages over solution-based sensors, such as the possibility of fabricating them onto almost any kind of substrate [91,92], their easy storage and portability [93], their regeneration and reusability [94], and the good chemical stability of luminophores in solid state [95]. Furthermore, they can be used for vapor/gas detection [96], an application field where the encapsulated probes are hardly used [97].

**Figure 6:**
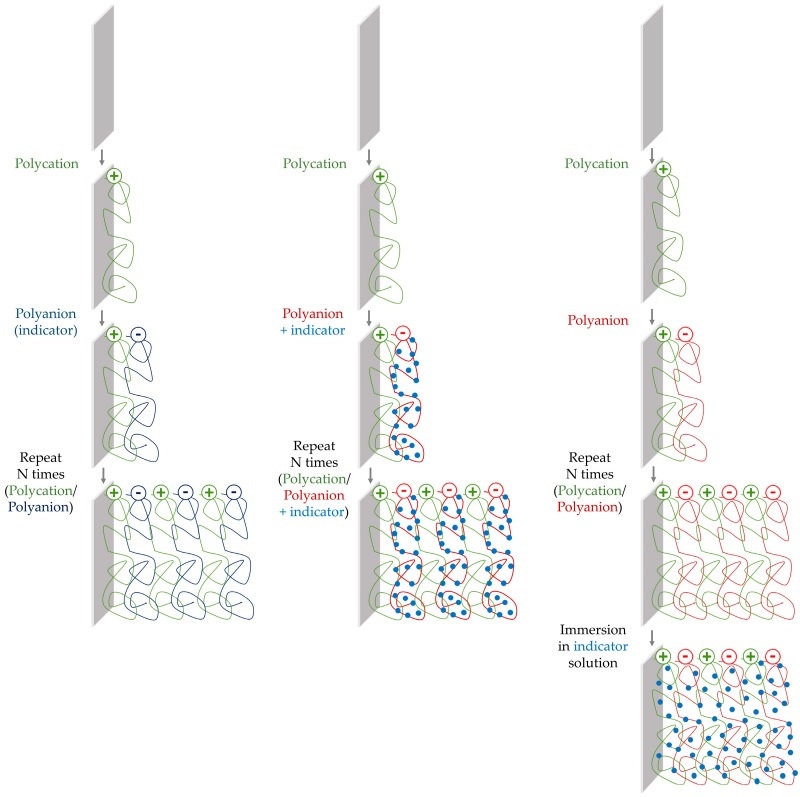
Schematic fabrication luminescent films: the non-neutral indicator is directly assembled into the film (**left** pathway), the neutral indicator is mixed, covalently linked, or entrapped into a charged material and then it is assembled into the coating (**central** pathway), or the fabricated film is immersed into a solution of the dye (**right** pathway).

The luminescent indicators can be entrapped in multilayer structures by different methods, which are displayed in Figure 6. If they are immobilized during the fabrication process, this can be done by direct assembly (in this case they are not neutral materials), or by mixing, covalently linking, or entrapping them inside charged materials. When the immobilization of the indicator occurs after the fabrication of the film, it is carried out by immersing it in a solution of the dye. Examples of these cases are explained in the following subsections.

### 3.1. LbL Luminescent Coatings for the Detection of Metal Ions

**Figure 7:**
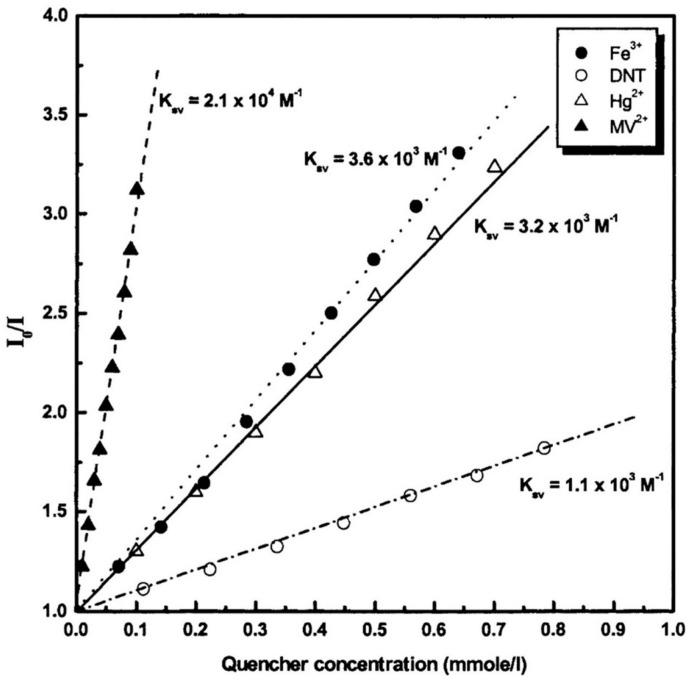
Stern−Volmer plots of multilayer films of PAH/PAA−HPTS as a function of different quencher concentrations. Reprinted with permission from [101]. Copyright © 2000, American Chemical Society.

A wide variety of indicators have been employed for the fabrication of luminescent films for the detection of metal ions, ranging from ligand-capped quantum dots to fluorescent conjugated polymers, including porphyrins and water-soluble dyes. To this latter category belongs 1-hydroxypyrene-3,6,8-trisulfonate, HPTS, a luminescent indicator widely used for pH monitoring [98,99]. For its deposition by the LbL technique, Lee and coworkers [100] attached it covalently to the polyanion PAA and, by using PAH as a cationic polyelectrolyte, deposited the multilayer structure [PAH/PAA-HPTS]_n_ onto glass slides. HPTS emitted luminescence at 485 nm when it was illuminated at 410 nm. The maximum of the luminescent peak decreased linearly, but with different quenching constants, in the presence of electron-deficient metal cations, such as Fe^3+^ and Hg^2+^, the nitro compound 2,4-dinitrotoluene, DNT, or the dicationic electron acceptor methyl viologen, MV^2+^ [101]. This can be observed in Figure 7, where the Stern–Volmer plots of multilayers of [PAH/PAA-HPTS] for each compound are displayed. Furthermore, this luminescence was not affected by other metal ions, for instance, Ba^2+^, Ca^2+^, K^+^, Zn^2+^, Cd^2+^, and Pb^2+^.

Different fluorescent probes for mercury(II) ion detection based on the water-soluble porphyrin 5,10,15,20-tetrakis(4-sulfonatophenyl)porphyrin (TPPS) were compared in [102]. PDDA was employed as a cationic polyelectrolyte and TPPS, PSS, or solutions with different ratios of TPPS:PSS were used as anionic counterparts. On one hand, it was observed that, when TPPS and PSS were co-deposited, giving rise to the structure (PDDA/TPPS:PSS)_n_, higher quantum yields were observed when the PSS:TPPS ratio increased from 1:1 to 1:100. On the other hand, when depositing TPPS alternately (i.e., PDDA/PSS/PDDA/TPPS)_n_), the amount of adsorbed porphyrin was higher than when as mixed with PSS, and a good quantum yield was also achieved. A detailed analysis of this structure demonstrated that films with one or two tetralayers were most suitable to be used as Hg(II) sensors, since they combined good optical properties with the lowest response time. In the case of (PDDA/PSS/PDDA/TPPS), the sensor was exposed to Hg(II) concentrations in the range between 3.3 × 10^−8^ and 3.3 × 10^−5^ M. For higher concentrations, a longer time was required to reach equilibrium as a consequence of the adsorption process of the analyte within the films.

**Figure 8:**
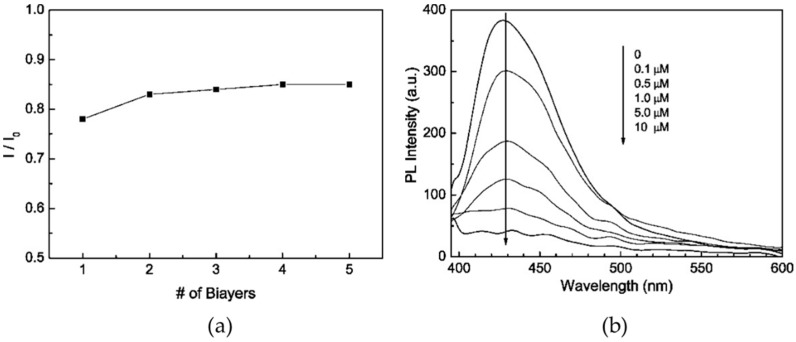
(**a**) Fluorescence response of (PDDA/PFPNa)_n_ structures upon addition of 0.1 μM Fe^3+^; (**b**) quenching of the fluorescent peak when the sensor (PDDA/PFPNa)_1_ is exposed to different Fe^3+^ concentrations. Reprinted with permission from [103]. Copyright: 2008, American Chemical Society.

Fluoropolymers have also attracted interest for the development of optical sensors because of their high luminescence quantum yields. For instance, poly(9,9-bis(3′-phosphatepropyl)fluorenealt-1,4-phenylene) sodium salt (PFPNa) was synthesized and deposited with PDDA by means of the LbL [103]. The PFPNa polymer had absorption and luminescence peaks at 364 and 410 nm, respectively, whose intensities were proportional to the pH of the aqueous solutions. The latter was also inversely proportional to Fe^3+^ concentration. Furthermore, the sensor sensitivity was demonstrated to be almost independent of the number of bilayers (Figure 8a), so the 1-bilayer coating was employed as the sensing structure. In this case, fluorescence was quenched 400-fold for 10 µM of Fe^3+^ concentration (Figure 8b), and the detection limit for this metal ion was 10^−7^ M. Another thin-film sensor that employs a fluoropolymer for metal ion detection is reported in [104].

**Figure 9:**
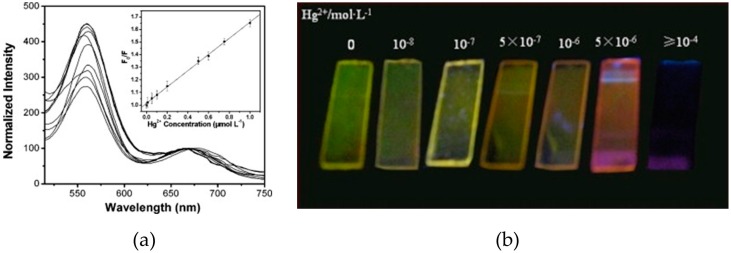
(**a**) Luminescence spectra of the bi-color film under exposure to different Hg^2+^ concentrations: 0 μM, 0.01 μM, 0.05 μM, 0.1 μM, 0.2 μM, 0.5 μM, 0.6 μM, 0.75 μM, and 1 μM. The inset shows the Stern–Volmer plot of the sensor; (**b**) Colors of the sensing films under exposure to different Hg^2+^ concentrations: 0 μM, 0.01 μM, 0.1 μM, 0.5 μM, 1 μM, 1.5 μM, and higher than 100 μM. Reprinted from [107] with permission from Elsevier.

Negatively charged mercaptosuccinic acid (MSA) capped CdTe QDs have been assembled with the cationic polyelectrolyte PDDA onto quartz slides by means of the LbL technique. These QDs exhibited a luminescent peak centered at 589 nm, whose intensity decreased proportionally with the increment of Hg(II) for concentrations ranging from 0.01 μM to 1 μM [105]. Hg(II) removal from the sensing film was possible by adding glutathione (GSH) to the sample solutions, which also led to the recovery of the initial luminescent intensity. In subsequent research, this multilayer structure was employed to monitor Hg^2+^ and Cu^2+^ synchronously [106]: despite both ions having quenched the luminescent intensity, the quenching constant of Hg^2+^ was higher than that of Cu^2+^. Finally, by cross-linking the outermost layer of PDDA/CdTe QDs multilayers by bovine serum albumin (BSA) [107], a bi-color film was developed: it exhibited two luminescent peaks centered at 553 nm (green) and 657 nm (red), with green being the dominant color. In the presence of Hg(II), the intensity of the first peak decreased (as shown in Figure 9a) and, for Hg(II) concentrations higher than 10^−6^ M, it was totally quenched, which made the sensing film change color from green to red. This color change was detectable by the naked eye, as can be seen in Figure 9b.

**Figure 10:**
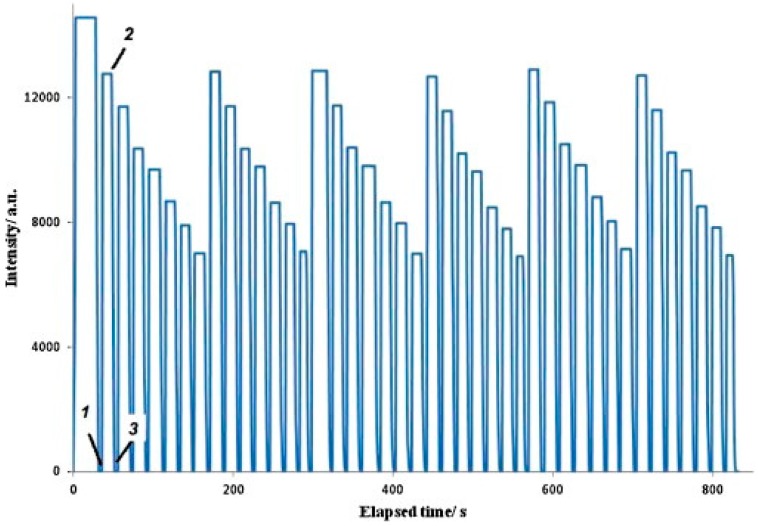
Steady-state fluorescence measurements over time (excitation 380 nm and emission 500 nm) of the dry optical fiber with six layers, followed by three cycles of Hg(II) aqueous solutions with the following concentrations: 0, 0.01, 0.05, 0.1, 0.799, 1.99, and 2.69 μM. (1) The fiber was immersed in water; (2) removed from water; and (3) immersed in Hg(II) 0.01 μM. Reprinted from [108] with permission from Elsevier.

Apart from CdTe QDs, carbon nanoparticles have also been employed for the fabrication of Hg(II)-sensitive luminescent films [108]. Their functionalization with PEG200 and N-acetyl-L-cysteine (NAC) enabled the carbon dots to be assembled with PEI onto the tapered tip of a 600 μm-core optical fiber by means of the LbL technique, as well as the detection of mercury ions. Although the fluorescence quenching mechanism of these sensors was not completely determined, it was likely to be due to the interaction between the –SH groups of NAC and Hg(II) ions. Sensing coatings from one to six bilayers of (PEI/carbon dots) were analyzed: all of them exhibited a reproducible and reversible behavior towards Hg(II) (see Figure 10 for the particular case of the six-layer structure), and it was found that an increase in the number of bilayers led to a decrease in the detection limit (0.1 μM Hg(II) for one bilayer and 0.01 μM Hg(II) for six bilayers) and an increase of the quenching constant. However, cross-sensitivity towards other metal ions was not studied.

**Figure 11:**
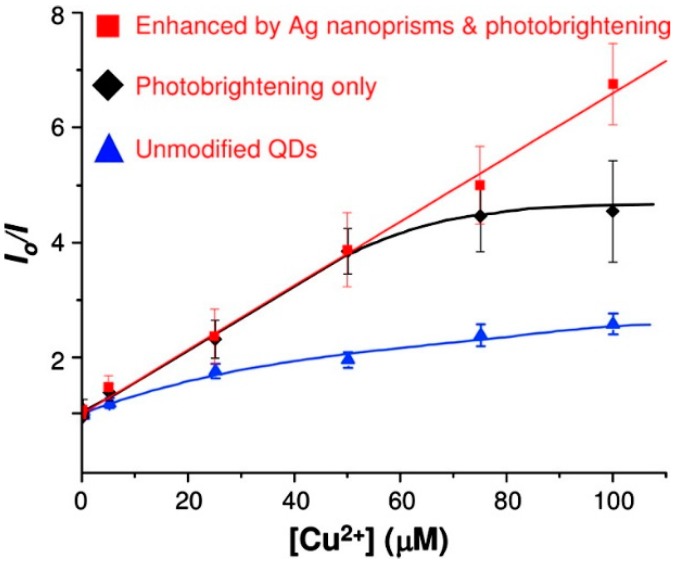
Stern−Volmer plot for the quenching of CdSe PL by Cu^2+^. The solid red squares (□), black diamonds (⧫), and blue triangles (▲) denote the CdSe QDs enhanced by both Ag nanoprisms and photobrightening, the photobrightened QDs, and the unmodified QDs, respectively. Note: the blue and black lines are added as a guide using fits to a third-order polynomial. The red line is a linear fit to the data. Reprinted from [109] with permission from Elsevier.

An ultrasensitive Cu(II) sensor was developed by taking advantage of the fluorescence enhancement produced by Ag nanoprisms onto 16-mercaptohexadecanoic acid (16-MHA) capped CdSe quantum dots (QDs) [109]. Si or glass slides were coated with a layer of silver nanoprisms, which was separated from the outer QDs layer by a (PDDA/PSS) spacer of the optimal thickness [110] fabricated by means of LbL. The Ag nanoprisms, together with a UV photobrightening process, enhanced the luminescence, which was selectively quenched by Cu^2+^ ions, with a detection limit as low as 5 nM. The enhancement produced by the photobrightening and the Ag nanoprisms is clearly observable in Figure 11.

Table 3 summarizes all the metal ion sensors analyzed in this section.

### 3.2. LbL Luminescent Coatings for Dissolved Oxygen Sensing

**Figure 12:**
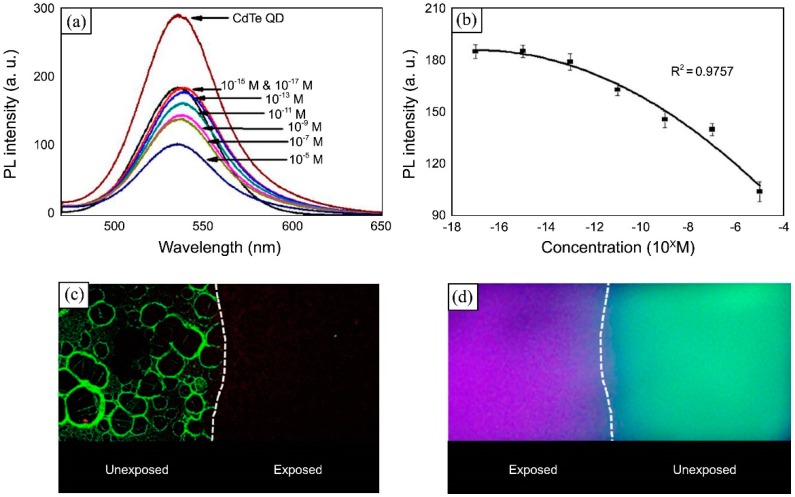
(**a**) Luminescent intensity of the film before and after incubation in solution of different phenylalanine concentrations; and (**b**) the corresponding calibration curve. After incubation and exposition to UV light, singlet oxygen is produced, which reacts with ascorbate, producing H_2_O_2_, which quenches the luminescence; (**c**) Microscopic image of the interface between a part exposed to phenylalanine (**right**) and a part unexposed (**left**); (**d**) UV image of the phenylalanine-exposed part (**left**), where only the UV light (excitation) is visible, and the unexposed part (**right**), where the luminescence (534 nm, green) is observable. Reprinted from [112] with permission from the Korean Chemical Society.

In neutral solutions singlet oxygen (^1^O_2_) reacts with ascorbate (AscH-) producing H_2_O_2_, which quenches the fluorescent emission of CdTe QDs [111]. Taking advantage of this reaction, a singlet oxygen sensitive coating was fabricated by means of the LbL technique [112]: first, a glass slide was coated with the base layers (PDDA/PAA)_3_/PDDA, onto which 10 bilayers of (CdTe QDs/PDDA) were deposited. With the aim of avoiding any interference between the CdTe QDs and the ascorbate, a spacer structure (PDDA/PAA)_2_/PDDA was introduced and, finally, two bilayers of PDDA/ascorbate were built. The sensing films were introduced in a phenylalanine solution, which produced singlet oxygen under UV illumination. Then, ^1^O_2_ reacted with ascorbate producing H_2_O_2_, which etched the QDs surface, giving rise to their luminescence quenching, as is shown in Figure 12. This sensing structure detected ^1^O_2_ concentration as low as 10^−15^ M. For each concentration, the intensity decreased for 5 min, when a steady stage was reached. This response time was thought to be due to the time required by H_2_O_2_ to etch the QDs [113].

Other dissolved oxygen sensors were developed by utilizing ruthenium porphyrins as sensitive materials. For instance, Grant and coworkers fabricated a self-referenced optical fiber sensor based on a polymer/polymer-dye multilayer structure, by combining the oxygen sensitive porphyrin bis(2,2′-bipyridine)′′-methyl-4-carboxybipyridine-ruthenium-N-succinimidyl-ester bis(hexafluoro-phosphate), Ru(bpy)_2_(mcbpy), with PAH and the reference dye, FITC, with the same cationic polyelectrolyte [114]. The multilayer architecture (PAH-Ru(bpy)_2_(mcbpy)/PSS)_10_ + {PAH-FITC/PSS)_5_ was built on the tip of a 400 μm-core optical fiber, which was connected to a two (200 μm) to one (400 μm) coupler. Under illumination at 450 nm, the sensing film exhibited two luminescent peaks, centered at 524 nm and 630 nm, which corresponded to the two dyes, FITC and Ru(bpy)_2_(mcbpy) respectively. The fluorescence peak ratio (I_630nm_/I_524nm_) changed from 0.82 to 0.75 under dissolved oxygen concentration variations from 0 to 1400 mM. In a subsequent study [115], the number of dye layers was increased up to 15, but no enhancement of the sensor performance was observed. 

A study of the adsorption of Ru(bpy) onto planar substrates by LbL was performed in [116]: this porphyrin was attempted to be assembled from a pure dye solution as well as from solutions of different dye-polyion concentrations. In the first case, the multilayer structure PEI/(PSS/PDDA)_2_/(PSS/Ru(bpy))_20_ was deposited onto glass slides. Despite Ru(bpy) being positively charged, it was observed that it was barely adsorbed to PSS and, what is more, it was desorbed when the substrates were immersed in the anionic solution. When mixing Ru(bpy) with PSS, sensing coatings with the structure PEI/(PSS/PDDA)_2_/(PSS-Ru(bpy)/PDDA)_20_ and different ratios (1:80, 1:40 and 1:20) of Ru(bpy):PSS were analyzed. As the Ru(bpy):PSS ratio increased, so did the fluorescence intensity. The fluorescence quenching of the sensor fabricated with the 1:20 (Ru(bpy):PSS) ratio exhibited a Stern–Volmer trend, being able to detect changes of less than 3% of the dissolved oxygen concentration in the range between 0 and 12 mg/L. This fact made it suitable for monitoring oxygen concentrations within biological environments. However, a further investigation [117] concluded that the best approach for adsorbing the luminescent dye to the substrate was not polyelectrolyte-dye mixing, but their covalent linkage: this bond prevented any kind of dye desorption when the substrate was immersed into the oppositely charged solution.

### 3.3. LbL Luminescent Coatings for Gaseous Oxygen Sensing

Another approach to immobilizing these ruthenium porphyrins into the multilayer structures consisted of the fabrication of the multilayer film and its further immersion in the dye solution, with the consequent diffusion of the indicator inside the coating. In [118], three kind of sensors were fabricated onto the tip of a 62.5 μm-core optical fiber. The first type consisted of a hygroscopic polymer membrane made of polyglutamic acid (PGA, anionic material) and poly-Lysine (cationic material). The second coating was a water absorbing polymer membrane composed of PAA (anionic polyelectrolyte) and PAH (cationic polyelectrolyte) and the third one, a porous composite membrane, was a multilayer structure of porous glass beads and PAH built onto a PAA layer. After the deposition of 50 bilayers of each structure onto the optical fiber tips, they were immersed in 80 mM Ru(bpy) solutions. When the sensors were illuminated at 450 nm, no phosphorescence was observed in the case of the water-absorbing polymer membrane, whereas the hygroscopic polymer membrane and the porous composite membrane exhibited a phosphorescent peak centered at 625 nm; the latter was the only one that was quenched when the sensor was exposed to 95–100% oxygen concentrations. Apart from the influence of the multilayer structure on the behavior of the sensors, the effect of the number of bilayers was also studied. It was found that a 125-bilayer structure did not show phosphorescence, which was attributed to the difficulty of introducing the ruthenium porphyrin in such a thick membrane. On the other hand, the five-bilayer sensor had a similar sensitivity to that of the 50-bilayers one, and it also demonstrated high resolution for low oxygen concentrations.

**Figure 13:**
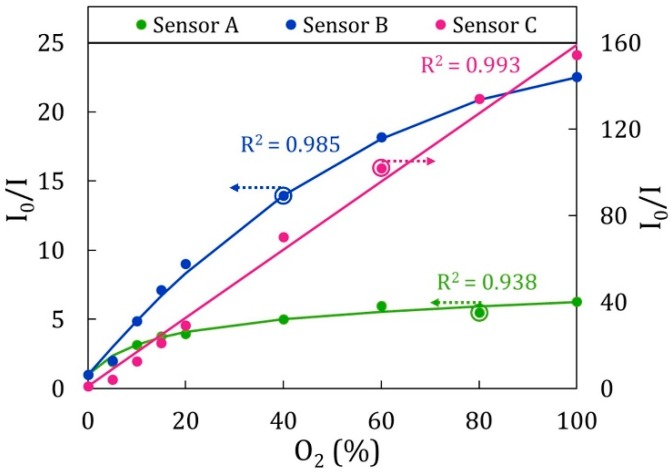
Calibration curves of (PDDA/Pt-TFPP_SDS_)_10_ (Sensor **A**), (PEI/Pt-TFPP_SDS_)_10_ (Sensor **B**), and (PAH/Pt-TFPP_SDS_)_10_ (Sensor **C**). Stern–Volmer plots of Sensors **A** and **B** are adjusted on the left axis, whereas that of Sensor **C** is adjusted on the right axis. Reprinted from [49] with permission from Elsevier.

All the ruthenium porphyrins employed up to now are water-soluble, which facilitates their assembly by means of the LbL technique. As is well known, this construction method requires all the materials to be present in water solutions for their deposition onto the substrates, so the water-insolubility of certain porphyrins can be an inconvenience. To overcome this fact and facilitate their deposition by the LbL technique, there exists the possibility of entrapping them into micelles [119]. Taking advantage of this method, the water-insoluble platinum(II)-5,10,15,20-tetrakis-(2,3,4,5,6-pentafluorphenyl) porphyrin, Pt-TFPP, was immobilized for the first time, employing the LbL technique in [120]: PAH was used as cationic polyelectrolyte and Sodium Dodecyl Sulfate (SDS) micelles, into which Pt-TFPP was entrapped, were employed as anionic counterpart. The multilayer coating formed by (PAH/Pt-TFPP_SDS_)_10_ was built onto the tip of a 400 μm-core optical fiber, which was connected to a 200 μm-core bifurcated fiber. Under illumination at 390 nm, the intensity of the luminescence peak at 650 nm decreased as the oxygen concentration increased, exhibiting a linear Stern–Volmer plot in the whole range of oxygen concentrations. A comparative study of three different polymeric matrices was carried out in [49], where the structures (PDDA/Pt-TFPP_SDS_)_10_, (PEI/Pt-TFPP_SDS_)_10_, and (PAH/Pt-TFPP_SDS_)_10_ were analyzed in detail. It was shown that the sensitivity was determined by the morphology of the coatings: the rougher the sensing film, the more sensitive the sensor, and the higher the range of oxygen concentrations able to detect the sensor. What is more, the sensors fabricated with PDDA and PEI did not exhibit linear calibration curves; their Stern–Volmer plots had two different quenching constants, indicating that the luminophore was not homogeneously distributed in the matrix. This fact can be seen in Figure 13 and in Table 4, where the calibration curves and the quenching constants of each sensor are displayed.

**Figure 14:**
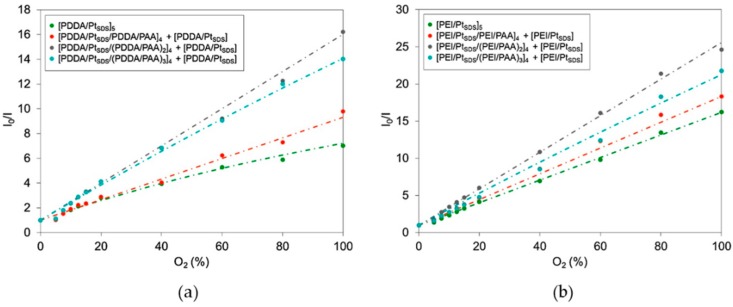

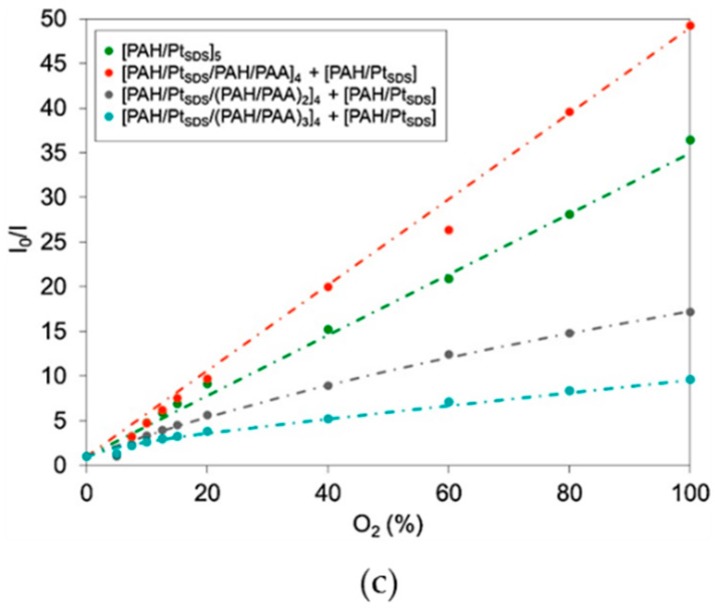
Stern–Volmer plots of the different sensors fabricated employing (**a**) PDDA, (**b**) PEI, and (**c**) PAH as cationic polyelectrolytes, and PAA as a spacer layer. In the cases of (**a**) PDDA and (**b**) PEI, the maximum of the sensitivity is achieved when luminescent layers are spaced by five layers of polyelectrolytes, whereas in the case of (**c**) PAH, only three layers of polyelectrolytes are necessary to achieve the maximum of the sensitivity. Reprinted from [48] with permission from Elsevier.

With the aim of avoiding self-quenching and enhancing the sensitivity of the sensors, the spacing distance between the luminescent films was increased by introducing PAA layers between the cationic ones [48]. This fact not only affected the sensitivities of the sensors, but also determined the distribution of the luminophores inside the multilayer structure. For instance, in the cases fabricating the sensors with PDDA or PEI, a higher number of spacing layers was needed than in the case of the sensors built with PAH to obtain linear calibration curves and the highest sensitivities. This is shown in Figure 14: for the sensors fabricated with PDDA or PEI, the highest sensitivities are obtained when the luminescent films (Pt_SDS_) are separated by five layers, P(+)/PAA/P(+)/PAA/P(+), where P(+) are PDDA and PEI, respectively. In the case of the sensors fabricated with PAH, only three spacing layers, PAH/PAA/PAH, are enough to reach the maximum sensitivity.

### 3.4. LbL Luminescent Films for Biosensing Applications

**Figure 15:**
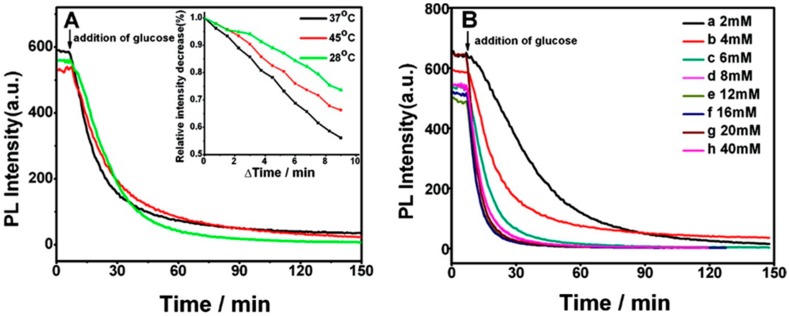

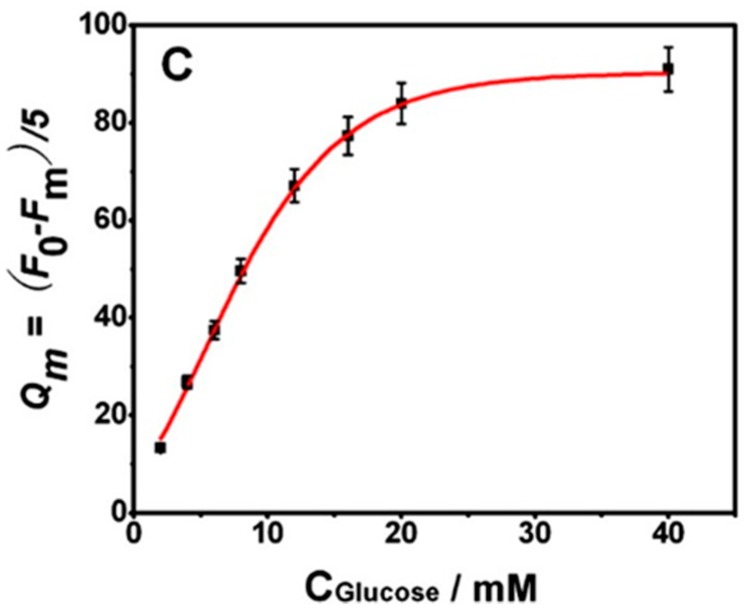
(**A**) Quenching of the luminescence peak centered at 630 nm of the multilayer structure (PAH/CdTe)_12_(PAH/PSS)_3_(PAH/GOD)_3_ when it is exposed to a 4 mM glucose solution at different temperatures. The time-dependent luminescence intensity of that peak during the first 9 min of the reaction for each temperature is shown in the inset. (**B**) Luminescence quenching of the same film for different glucose concentrations: (**a**) 2, (**b**) 4, (**c**) 6, (**d**) 8, (**e**) 12, (**f**) 16, (**g**) 20, and (**h**) 40 mM over 150 min; (**C**) quenching rate (Q_m_) of the sensor over 5 min as a function of the glucose concentration. *F*_0_ and *F*_m_ correspond to the luminescence intensity in the absence (*F*_0_) and presence (*F*_m_) of glucose. All measurements were carried out in a 20 mM phosphate buffer at pH 7.4. Copyright: 2009, American Chemical Society.

Li et al. used the multilayer structure (PAH/CdTe QDs)_x_(PAH/PSS)_3_(PAH/GOD)_y_ to determine the concentration of blood glucose in real serum samples with good reproducibility and accuracy [113]; the GOD enzyme catalyzed the reaction between oxygen and glucose, producing H_2_O_2_, which generated defects on the surface of the QDs, quenching their luminescence. Under illumination at 380 m, the initial structure (PAH/CdTe QDs)_12_(PAH/PSS)_3_(PAH/GOD)_3_ showed a luminescence peak centered at 630 nm. The luminescent properties of this coating were analyzed for different temperature and pH values ranging from 28 to 45 °C, and from 6 to 9, respectively. In the case of temperature, the largest quenching rate was obtained for 37 °C, whereas a pH value of 7.4 was chosen as optimal. Figure 15 shows the luminescence quenching of that structure at different temperatures (A), upon different glucose concentrations (B), and the absolute quenching rate of this structure (C). 

**Figure 16:**
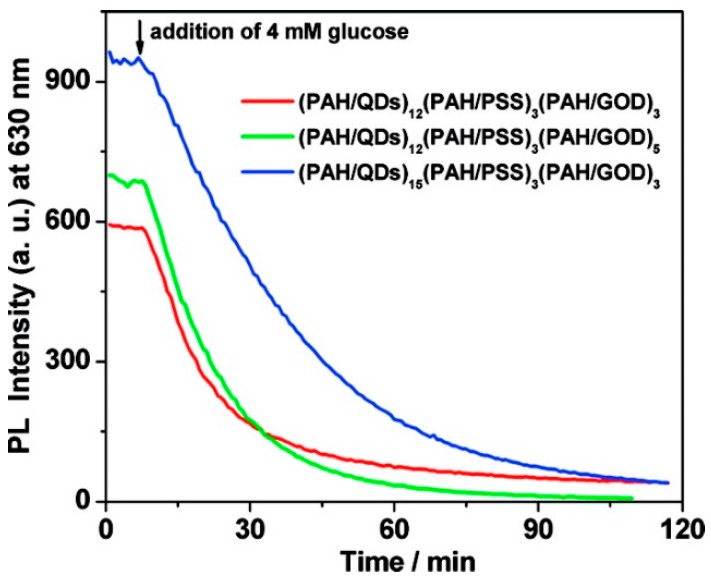
Luminescence quenching at 630 nm (λ_ex_ = 380 nm) when different structures of (PAH/CdTe QDs)_x_(PAH/PSS)_3_(PAH/GOD)_y_ were exposed to 4 mM glucose. All measurements were carried out at 37 °C in a 20 mM phosphate buffer at pH 7.4. Copyright: 2009, American Chemical Society.

Under those conditions (37 °C and pH 7.4), the response upon addition of 4 mM glucose of three different structures (as shown in Figure 16) was studied with the aim of optimizing the number of PAH/CdTeQDs and PAH/GOD bilayers. For a given number of PAH/GOD bilayers (in this case, three), it was observed that the quenching rate of the sensor decreased when the increase in the number of QDs layers was limited to 12. For this number of PAH/CdTe QDs bilayers, the quenching constant increased linearly with the number of enzyme layers as a consequence of the good permeability of the GOD layers towards glucose. The PAH/PSS_3_ spacer was introduced in order to avoid any kind of influence of the GOD-glucose reaction on the QDs. The structure (PAH/CdTe QDs)_12_(PAH/PSS)_3_(PAH/GOD)_5_ was chosen as optimal for determining the blood glucose concentration in serum samples: it exhibited linear luminescence quenching in the glucose concentration range between 0.5 and 16 mM, with 0.5 mM being the detection limit. Furthermore, no sample pre-treatment was needed.

Since it was isolated, graphene and some related structures have been employed for diverse sensing applications [121]. Graphene oxide multilayer arrays were assembled by means of the LbL technique by Jung et al. [122] for the fabrication of aptasensor microarrays. These multilayers were prepared by assembling oppositely charged graphene oxide sheets: negatively charged ones (GO^−^) were prepared by introducing COOH groups, while positively charged sheets (GO^+^) were obtained thanks to the NH_2_ groups. An aminated glass slide was coated with 10 bilayers of (GO^−^/GO^+^), and then a FAM-labeled thrombin aptamer was immobilized on them. The FAM luminescence, centered at 530 nm, was quenched by graphene oxide due to the high energy transfer between the dye and graphene. In the presence of thrombin, fluorescence was recovered owing to the high affinity between aptamers and thrombin. On the other hand, other analytes such as bovine serum albumin (BSA), streptavidin (STA), glucose, and human immunoglobulin (IgG) antibody did not alter the quenched fluorescence of FAM-aptamer-labeled GO multilayers, probing the high specificity of the aptamer-based sensor. For the particular case of 10 bilayers of (GO^−^/GO^+^) and 2 μM aptamer concentration, the fluorescence intensity from FAM was quenched over 85% and the detection limit for thrombin was 0.001 nM, exhibiting 30-fold higher sensitivity than the solution-based graphene FRET aptasensor [123]. Furthermore, this sensor was reused four times by simply cleaning it with distilled water.

## 4. Conclusions

It is obvious that luminescence has become a powerful detection mechanism for biological and environmental applications. This sensing method also takes advantage of the wide variety of sensitive luminophores that exists: fluoropolymers, water-soluble and non-soluble porphyrins, or semiconductor quantum dots, among others. These materials can be encapsulated in multilayer shells for their utilization in solution-based probes, or they can be entrapped inside nanostructured films and used as solid-state sensors. In both cases, the LbL nano-assembly technique permits us to tailor the properties of the sensors at the nanometer scale, making feasible the fabrication of custom-made devices that not only exhibit good photostability, but also high selectivity and sensitivity for almost any kind of sensing applications.

The remarkable characteristics of the sensors exposed in this study suggest that the combination of luminescence and the LbL nano-assembly technique is a promising approach for the fabrication of sensing devices for real applications. Environmental and biosensing purposes are probably the most encouraging fields owing to the facility for fabricating arrays of sensor capable of detecting several analytes with a single measurement. Furthermore, a real solution for sensing applications in hazardous environments can be obtained by combining luminescence and the Layer-by-Layer technique with the unique properties of optical fibers.

## Figures and Tables

**Table 1:** Encapsulated sensors for ions detection.

Analyte	Sensitive Indicator	Reference Indicator	Capsule	Detection Range	LOD	Ref.
K^+^	PBFI	-	(PSS/PAH)_5_	0–45 mM		[64]
K^+^	PBFI	FluoSpheres	(PAH/PSS)_4_PAH	0–282 mM		[84]
K^+^	PBFI	-	2, 3, and 5 bilayers of {PSS/PDDA}	0–45 mM	-	[80]
K^+^	PBFI	Europium FluoSpheres	(PAH/PSS)_4_PAH	0–120 mM	1 mM	[85]
K^+^	PBFI	Europium FluoSpheres	(PAH/PSS)_4_PAH	0–300 mM	1.2 mM	[86]
Na^+^	SBFI	-	2, 3, and 5 bilayers of {PSS/PDDA}	0–100 mM	-	[54]
Cu^2+^, Ag^+^	CdSe/ZnSe QDs	NaYF4:Ce,Tb rare-earth nanocrystals	PSS/PAH	0–35 μM Cu^2+^ 0–90 μM Ag^+^	-	[83]
Pb^2+^	CdSe/CdS QDs	-	chitosan/xylenol orange	0.05–6 μM	20 nM	[59]

**Table 2:** Encapsulated sensors for dissolved oxygen detection.

Sensitive Indicator	Reference Indicator	Capsule	Detection Range	LOD	Ref.
Ru(dpp)	green polystyrene FluoSpheres	{PAH/PSS}_3_	0–1500 mM	-	[85]
Ru(dpp)	carboxylate-modified nanospheres	(PAH/PSS)_3_	ON/OFF probe	-	[74]
Ru(dpp)	-	(PSS/PAH)_4_/PSS	0–0.6 mM	-	[87]
Ru(bpy)	FITC	(PSS/PAH-FITC)_5_/PSS		-	[52]
Ru(bpy)	FITC	(PSS/PDDA)_5_/PSS	ON/OFF probe	-	[75]
[Ru(Ph_2_phen)_3_]^2+^	carboxylate-modified FluoSpheres	{PAH/PSS}_3_	0–1.5 mM	-	[88]
PdTCPP	carboxylate-modified FluoSpheres	[PDDA/PSS]_10_	0–250 µM	7.62 µM	[58]

**Table 3:** Luminescent films fabricated by LbL for ions detection.

Analyte	Sensitive Indicator	Sensing Film	Detection Range	LOD	Cross-Sensitivity	Ref.
Fe^3+^, Hg^2+^	HPTS	[PAH/PAA-HPTS]n	0–0.5 mM Fe^3+^ 0–1 mM Hg^2+^	1.28 ppm Fe^3+^ 1.79 ppm Hg^2+^	-	[100,101]
Hg^2+^	TPPS	(PDDA/PSS/PDDA/TPPS)	0–3.3 × 10^−5^ M	<3.3 × 10^−8^ M	Cd^2+^, Pb^2+^, Cu^2+^	[102]
Hg^2+^	PPESO_3_	(PDDA/PPESO_3_)_3_	0–1 mM	10^−7^ M	Fe^3+^, Al^3+^	[104]
Fe^3+^	PFPNa	(PDAC/PFPNa)_1_	0–10 μM	10^−7^ M	-	[103]
Hg^2+^	MSA-capped CdTe QDs	(PDDA/QDs)_10_	0–1 μM	<10^−8^ M	Cu^2+^, Ag1+	[105]
Cu^2+^, Hg^2+^	MSA-capped CdTe QDs	(PDDA/QDs)_5_	0–1 μM Cu^2+^ 0–0.5 μM Hg^2+^	<10^−8^ M Cu^2+^ <5 × 10^−9^ M Hg^2+^	High concentrations of Ni^2+^, Cr^3+^, Au^3+^, Ag^+^	[106]
Hg^2+^	MPA-capped CdTe QDs	(PDDA/QDs)5/PDDA/PSS/PDDA/(QDs)5/BSA	0.01–1 μM	4.5 × 10^−9^ M	-	[107]
Hg^2+^	Carbon dots	(PEI/Carbon dots)_1-6_	0.01–2.69 μM for (PEI/Carbon dots)_6_	10^−8^ M for (PEI/Carbon dots)_6_	-	[108]
Cu^2+^	(16-MHA) capped CdSe QDs	Ag NPs/(PDADMAC/PSS)/QDs	0–100 μM	5 × 10^−9^ M	-	[109]

**Table 4:** Quenching constants and calibration curves of the three oxygen sensors fabricated employing PDDA, PEI, or PAH as cationic polyelectrolytes. Data obtained from [48].

	Stern–Volmer Constants				Mathematical Model
	f_1_	K_SV,1_	f_2_	K_SV,2_	
[PDDA/Pt_SDS_]_5_	0.957	0.0898	0.043	0.0001	$\frac{I_{0}}{I}={\left(\frac{0.957}{1+0.0898\cdot [O_{2}]}+\frac{0.043}{1+0.0001\cdot [O_{2}]}\right)}^{-1}$
[PEI/Pt_SDS_]_5_	0.9939	0.1526	0.0061	0.085	$\frac{I_{0}}{I}={\left(\frac{0.9939}{1+0.1526\cdot [O_{2}]}+\frac{0.0061}{1+0.085\cdot [O_{2}]}\right)}^{-1}$
[PAH/Pt_SDS_]_5_	1	0.34	0	0	$\frac{I_{0}}{I}=1+0.34\cdot [O_{2}]$
